# Cavity-mediated coupling between local and nonlocal modes in Landau polaritons

**DOI:** 10.1515/nanoph-2025-0442

**Published:** 2025-11-18

**Authors:** Sae R. Endo, Dasom Kim, Shuang Liang, Geon Lee, Sunghwan Kim, Alan Covarrubias-Morales, Minah Seo, Michael J. Manfra, Dukhyung Lee, Motoaki Bamba, Junichiro Kono

**Affiliations:** Smalley–Curl Institute, 3990 Rice University, Houston, TX 77005, USA; Department of Applied Physics and Physico-Informatics, Keio University, Yokohama 223-8522, Japan; Department of Electrical and Computer Engineering, 3990 Rice University, Houston, TX 77005, USA; Department of Physics and Astronomy, Purdue University, West Lafayette, IN 47907, USA; Sensor System Research Center, Korea Institute of Science and Technology (KIST), Seoul 02792, Republic of Korea; Department of Physics, Ulsan National Institute of Science and Technology (UNIST), Ulsan 44919, Republic of Korea; Applied Physics Graduate Program, Smalley–Curl Institute, 3990 Rice University, Houston, TX 77005, USA; Department of Physics, Sogang University, Seoul, 04107, Republic of Korea; School of Applied and Engineering Physics, Mohammed VI Polytechnic University (UM6P), Ben Guerir 43150, Morocco; Department of Physics, Graduate School of Engineering Science, Yokohama National University, Yokohama 240-8501, Japan; Institute for Multidisciplinary Sciences, Yokohama National University, Yokohama 240-8501, Japan; Rice Advanced Materials Institute, 3990 Rice University, Houston, TX 77005, USA; Department of Physics and Astronomy, 3990 Rice University, Houston, TX 77005, USA; Department of Materials Science and NanoEngineering, 3990 Rice University, Houston, TX 77005, USA

**Keywords:** multimode coupling, magnetoplasmons, Landau polaritons

## Abstract

The multimode ultrastrong coupling (USC) regime has emerged as a novel platform for accessing previously inaccessible phenomena in cavity quantum electrodynamics. Of particular interest are cavity-mediated correlations between local and nonlocal excitations, or equivalently, between modes at zero and finite in-plane momentum, which offer new opportunities for controlling light–matter interactions across space. However, direct experimental evidence of such interactions has remained elusive. Here, we demonstrate nonlocal multimode coupling in a Landau polariton system, where cavity photons simultaneously interact with the zero-momentum cyclotron resonance and finite-momentum magnetoplasmons of GaAs two-dimensional electron gas. Our slot cavities, with their subwavelength mode volumes, supply in-plane momentum components that enable the excitation of finite-momentum matter modes. Terahertz time-domain magnetospectroscopy measurements reveal a clear splitting of the upper-polariton branch, arising from hybridization between magnetoplasmon modes and the cavity–cyclotron-resonance hybrids. Extracted coupling strengths confirm USC of the cyclotron resonance and strong coupling of the magnetoplasmon modes to the cavity field, respectively. The experimental results are well captured by the multimode Hopfield model and finite-element simulations. These findings establish a pathway for engineering multimode light–matter interactions involving zero- and finite-momentum matter modes in the USC regime.

## 1 Introduction

Vacuum–matter interactions in cavities are currently explored in diverse systems both for controlling material properties without any external driving field and for applications in quantum information technologies. The ultrastrong coupling (USC) of vacuum–matter arises when the vacuum Rabi frequency becomes a significant fraction of the bare frequencies of vacuum and matter at zero detuning (*ω*_0_), i.e., *g*/*ω*_0_ ≥ 0.1, where *g* is the coupling strength [1], [2]. This regime gives rise to exotic phenomena via the non-negligible contribution of the counter-rotating terms in the Hamiltonian, such as quantum squeezing in the ground state [3], [4], the vacuum Bloch–Siegert shift [5], the Dicke superradiant phase transition [6], [7], cavity-mediated superconductivity [8], and modifications of the quantum Hall effect [9], [10]. The USC regime has been realized in Landau polaritons [5], [11], [12], [13], [14], [15], [16], [17], [18], [19], [20], [21], phonon polaritons [22], [23], [24], [25], and magnon polaritons [7], [26], [27]. Among these systems, Landau polaritons offer exceptional tunability through externally applied magnetic fields.

Recently, attention has expanded toward the multimode USC regime, where a matter excitation ultrastrongly couples simultaneously with multiple cavity modes, or conversely, a single cavity mode interacts with multiple matter excitations. This regime is not only a natural extension of single-mode USC but also introduces fundamentally new physics due to the nontrivial interplay between cavity modes or between matter excitations [20], [25], [28], [29], [30], [31]. For example, correlations between bare excitations emerge due to the significant contribution of the counter-rotating terms, which do not exist in the absence of ultrastrong light–matter interactions. Very recently, matter–mediated photon–photon correlations [31] and cavity-mediated superthermal phonon–phonon correlations [25] have been realized by leveraging the large dipole moment of Landau-level transitions and the subwavelength light confinement of metamaterial cavities, respectively.

Furthermore, nonlocal, i.e., *k* ≠ 0 aspects of USC are also drawing increasing interest [32], where *k* is the in-plane wave vector of matter excitation. In particular, the possibility of mediating long-range nonlocal correlations through the vacuum field opens a new direction in controlling collective matter excitations across space. Therefore, it would be particularly interesting to explore hybrid quantum states that bridge local and nonlocal matter excitations – coupling between *k* = 0 and finite *k* modes mediated by a cavity mode. While recent studies have explored multimode USC involving finite *k* excitations [19], [30], [32], [33], direct observation of mode-resolved anticrossings between individual modes has so far remained elusive. To further deepen our understanding of multimode USC, it is desirable to investigate systems where such interactions can be more clearly resolved.

Here, we report spectroscopic evidence for coupling between a local mode and a nonlocal mode, through simultaneous USC and strong coupling (SC) with a single cavity mode, in a Landau polariton system. We fabricated an array of slot cavities on a two-dimensional electron gas (2DEG), and its microstructure allowed cavity photons to couple with finite-*k* plasmon modes of the 2DEG by providing in-plane momentum. In a magnetic field, we experimentally observed SC of magnetoplasmon (MP) modes and the cavity mode, as well as USC between the cyclotron resonance (CR) of the 2DEG and the cavity mode. This multimode hybridization resulted in a clear splitting of the upper-polariton (UP) branch, which can be described by the multimode Hopfield model. Simulated transmission spectra using the finite-element method exhibited similar behavior, confirming the effect of finite-*k* MP modes. Our findings highlight a new platform where local and finite-*k* matter excitations can be coupled through a cavity mode, offering a perspective on momentum-resolved multimode interactions in the USC regime.

## 2 Results

**Figure 1:**
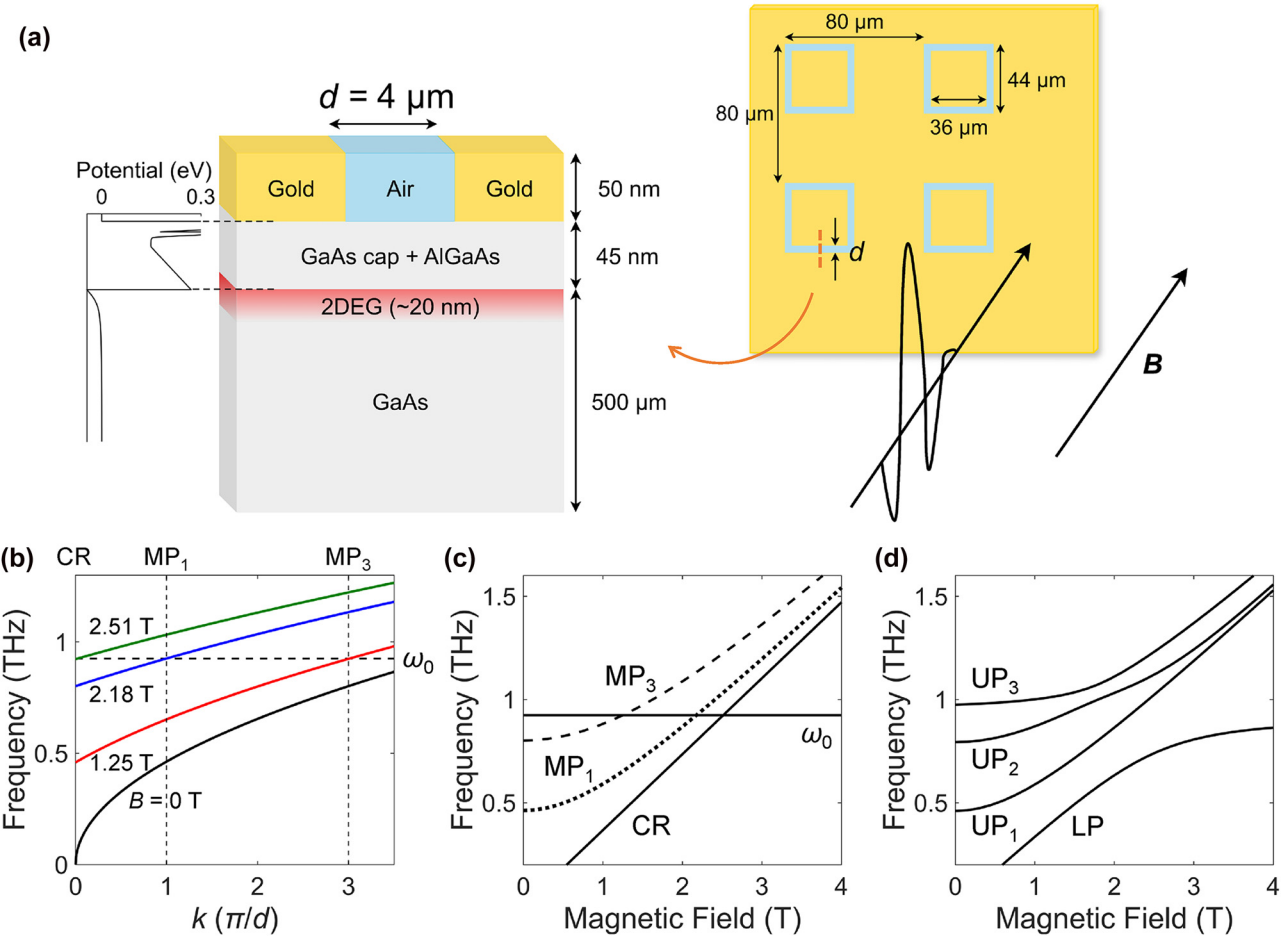
A multimode coupled system involving local and nonlocal matter excitations, and a cavity mode. (a) Schematic of the GaAs 2DEG–slot system. 2DEG: two-dimensional electron gas. (b) Dispersion relations of plasmons at 0 T (black) and magnetoplasmons at 1.25 T (red), 2.18 T (blue), and 2.51 T (green), corresponding to zero-detuning magnetic fields of MP_1_, MP_3_, and CR to *ω*_0_. *ω*_0_: bare cavity frequency. (c), (d) Illustration of the multimode coupling of CR, MP_1_, and MP_3_ to the cavity mode. (c) Uncoupled case. (d) Coupled case. The multimode coupling leads to the formation of one lower polariton (LP) and three upper polaritons (UP_1_, UP_2_, and UP_3_).

Providing in-plane momentum for light–matter interactions has long been achieved using periodic lattice structures [34], [35], [36], [37], [38], [39], via patterning 2DEGs [17], [19], [33], [40], [41], [42], [43], [44], [45] or by confining light in a small mode volume [32], [46]. To implement this mechanism, we fabricated an array of slot cavities on a GaAs 2DEG using standard photolithography and lift-off techniques. The 2DEG was present about 45 nm below the slot, ensuring strong overlap with the electromagnetic fields of the cavity mode; see Figure 1(a). The electron density and mobility were *n*_e_ = 3.6 × 10^11^ cm^−2^ and *μ*_e_ = 1.2 × 10^6^ cm^2^/(V s), respectively, obtained through van der Pauw measurements. The electron cyclotron mass was found to be m* = 0.076*m*_0_ through terahertz (THz) magnetospectroscopy measurements, where *m*_0_ is the free electron mass in vacuum. The loop length of slots was tuned to have a resonance frequency in the THz frequency range. The bare cavity frequency, *ω*_0_, was at 0.925 THz. The width of the slot, *d*, was 4 μm to confine light inside the cavity, providing cavity photons with finite in-plane momentum. Although the periodic structure also provided finite in-plane momentum, its frequency was too low compared with the cavity mode. Therefore, we consider only the *k* components generated by the individual slots.

As a finite-*k* matter excitation, we had the plasma oscillations of the 2DEG. The 2D plasma frequency in the long-wavelength limit is given by [47]

(1) $$\omega_{\text{p}}\left(k\right)=\sqrt{\frac{ke^{2}n_{\text{e}}}{2m^{*}\varepsilon_{0}\varepsilon_{\text{r}}}},$$

where *ɛ*_0_, *ɛ*_r_, and *e* are the vacuum permittivity, the effective dielectric constant of the surrounding medium (*ɛ*_r_ = 6.98), and the elementary charge, respectively; see Figure 1(b) (black solid line). The slot can provide the in-plane momentum *k* = *nπ*/*d*, where *n* is restricted to odd integers due to the symmetry of the electric field [19], [41]. While higher-order terms are possible, they are typically weaker and more difficult to observe. Based on our experimental results, we focus on the lowest two components, corresponding to the *n* = 1 and 3 modes. However, even these modes alone do not provide sufficient momentum to match the plasma resonance to the cavity frequency *ω*_0_, resulting in finite detuning, i.e., there is no intersection between the cavity mode and the *n* = 1 and 3 MP modes at *k* ≤ 3*π*/*d*.

Another matter excitation in our system was the CR of the 2DEG, arising from transitions between adjacent Landau levels at *k* = 0, which can be tuned by a perpendicular magnetic field, *B*, through the cyclotron frequency *ω*_c_ = *eB*/m*. In the presence of *B*, the 2D plasma excitations evolve into MPs, whose dispersion is given by [48], [49]

(2) $$\omega_{\text{MP}}^{2}\left(k\right)=\omega_{\text{p}}^{2}\left(k\right)+\omega_{\text{c}}^{2}.$$

As shown in Figure 1(b) (colored lines), the entire dispersion curve blue-shifts as the magnetic field increases. The colored curves represent the MP dispersion at various magnetic field strengths, with the red (at 1.25 T), blue (at 2.18 T), and green lines (at 2.51 T) indicating the zero-detuning conditions to the cavity mode. These field-tuned resonances can satisfy the momentum matching condition for *n* = 3 mode (MP_3_), *n* = 1 mode (MP_1_), and CR, respectively, enabling the exploration of hybridization of *k* = 0 and *k* ≠ 0 matter excitations through the cavity mode.

Figure 1(c) plots the bare matter excitation frequencies and the cavity frequency as a function of *B*. In this uncoupled situation, the CR and MP modes cross the cavity mode, showing three zero-detuning points, as expected from Figure 1(b). However, when the CR and MP modes are coupled with the cavity mode, they are expected to exhibit an anticrossing behavior at each zero-detuning point; see Figure 1(d). The multimode coupling gives rise to one lower polariton (LP) and three upper polariton branches (UP_1_, UP_2_, and UP_3_).

**Figure 2:**
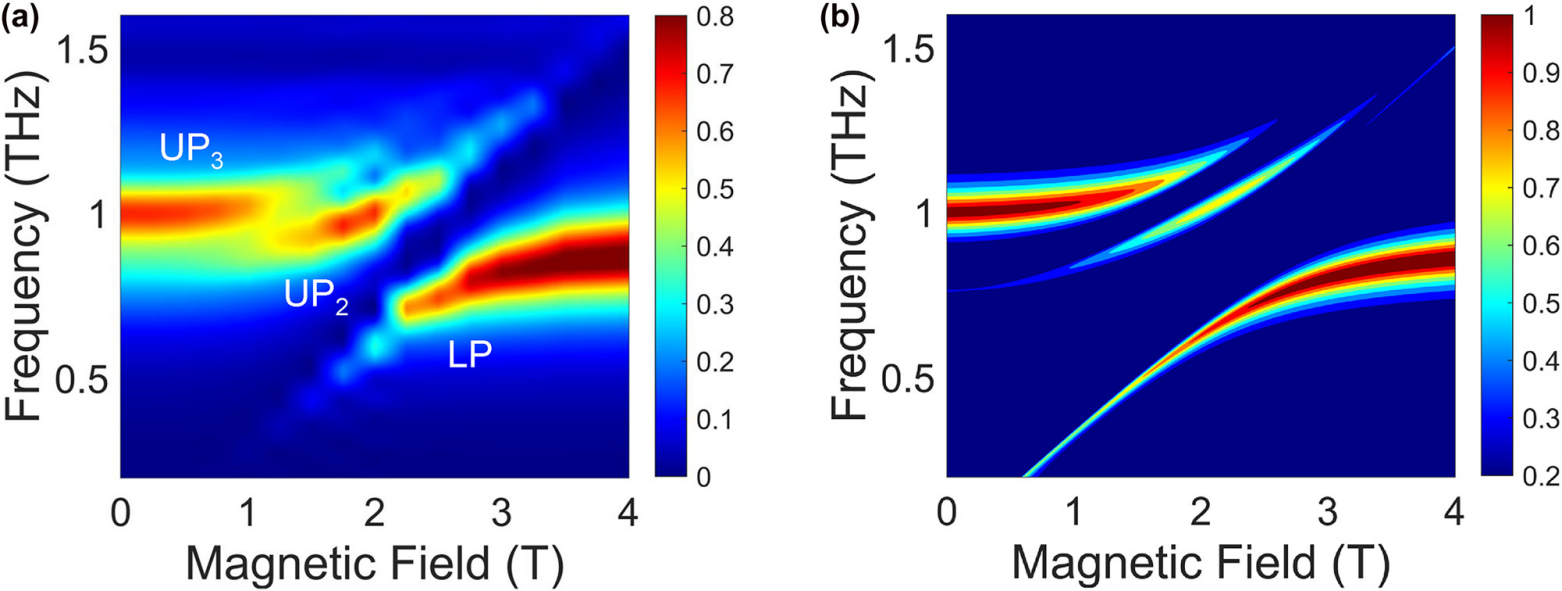
Spectroscopic evidence for the multimode coupling. (a) Experimental color map of transmittance as a function of frequency and magnetic field. The upper polariton is split into two parts, corresponding to UP_2_ and UP_3_ in Figure 1(d). (b) Theoretical color map of transmittance based on the multimode Hopfield model.

To confirm our model, we performed THz time-domain magnetospectroscopy measurements on the coupled system. THz pulses were generated and detected using InGaAs photoconductive antennas fiber-coupled to an Er-doped fiber laser with a center wavelength of 1,550 nm and repetition rate of 80 MHz. The electric field strength was sufficiently low to prevent any nonlinear effects. Measurements were conducted at 1.6 K in a magneto-optical cryostat operating in the Faraday geometry. Time-domain signals of the samples and reference (bare GaAs 2DEG) were Fourier transformed to obtain transmission spectra from 0.2 THz to 1.6 THz. Further details of the experimental setup are provided in the Supplementary Materials. Figure 2(a) shows a color map of transmittance as a function of frequency and magnetic field. At around 2.50 T, an anticrossing behavior is observed, arising from the USC between the cavity mode and the CR of the 2DEG at *k* = 0. At around 1.25 T, an additional splitting of the UP branch is clearly observed. This is evidence of SC of the MP_3_ with the cavity mode having *k* = 3*π*/*d*, induced by the confinement in the 4 μm slot. The split upper polaritons can be interpreted as UP_2_ and UP_3_ in Figure 1(d). UP_1_ is absent in the experimental data due to the THz spectra reflecting the cavity-like components of the polaritons, as well as the proximity of UP_1_ to the CR at finite fields.

To theoretically explain the coupling between a cavity mode, CR, and MP modes, we introduce a multimode Hopfield model. By adding the MP modes to the full Hamiltonian of Landau polaritons presented in ref. [5] while considering only a single cavity mode, the multimode Hamiltonian can be written as

(3) $$\begin{array}{ll} \frac{\hat{H}}{\hbar } & =\underset{\xi =\pm }{\sum }\omega_{0}{\hat{a}}_{\xi }^{†}{\hat{a}}_{\xi }+\omega_{\text{c}}{\hat{b}}^{†}\hat{b}+\underset{n}{\sum }\omega_{{\text{MP}}_{n}}{\hat{c}}_{n}^{†}{\hat{c}}_{n}\\ & \;+i\left[\left(\bar{g}{\hat{b}}^{†}+\underset{n}{\sum }{\bar{g}}_{n}{\hat{c}}_{n}^{†}\right)\left({\hat{a}}_{+}+{\hat{a}}_{-}^{†}\right)\right.\\ & \;\left.-\left(\bar{g}\hat{b}+\underset{n}{\sum }{\bar{g}}_{n}{\hat{c}}_{n}\right)\left({\hat{a}}_{-}+{\hat{a}}_{+}^{†}\right)\right]\\ & \;+D\left({\hat{a}}_{-}+{\hat{a}}_{+}^{†}\right)\left({\hat{a}}_{+}+{\hat{a}}_{-}^{†}\right), \end{array}$$

where *ℏ* is the reduced Planck constant, ${\hat{a}}^{†}$ $\left(\hat{a}\right)$ in the first term is the creation (annihilation) operator for the cavity photons of CR-active (*ξ* = +) and CR-inactive (*ξ* = −) circularly polarized modes, the second term represents the energy of the collective CR excitation of the 2DEG, ${\hat{b}}^{†}$ $\left(\hat{b}\right)$ being its creation (annihilation) operator, MP modes of different orders *n* = 1, 3 are introduced in the third term, where ${\hat{c}}_{n}^{†}$ $\left({\hat{c}}_{n}\right)$ and $\omega_{{\text{MP}}_{n}}$ are the corresponding creation (annihilation) operator and resonance angular frequency, and the fourth term is the light–matter interaction term considering the coupling of CR and all MP modes with the single cavity mode.

Here, $\bar{g}$ and ${\bar{g}}_{n}$ are the coupling strengths of CR and the *n*-th MP mode with the cavity mode, respectively. Using the zero-detuning coupling strengths, *g* and *g*_*n*_, which are independent of the external *B*, $\bar{g}$ and ${\bar{g}}_{n}$ can be written as

(4) $$\bar{g}=\sqrt{\frac{\omega_{\text{c}}}{\omega_{0}}}g,$$

(5) $${\bar{g}}_{n}=\sqrt{\frac{\omega_{{\text{MP}}_{n}}}{\omega_{0}}}g_{n}.$$

The coefficient *D* of the diamagnetic term is

(6) $$D=\frac{{\bar{g}}^{2}}{\omega_{\text{c}}}+\underset{n}{\sum }\frac{{\bar{g}}_{n}^{2}}{\omega_{{\text{MP}}_{n}}}=\frac{g^{2}+\underset{n}{\sum }g_{n}^{2}}{\omega_{0}}.$$

The coupling strengths appear in the relative permittivity as

(7) $$\begin{array}{ll} \varepsilon \left(\omega \right) & =\varepsilon_{\text{bg}}-\frac{\omega_{\text{pl}}^{2}}{\omega \left(\omega -\omega_{\text{c}}+\frac{i}{\tau }\right)}\\ & \;-\underset{n}{\sum }\frac{\omega_{{\text{pl}}_{n}}^{2}}{\omega \left(\omega -\omega_{{\text{MP}}_{n}}+\frac{i}{\tau_{n}}\right)}, \end{array}$$

where the plasma frequencies of CR and *n*-th MP mode, *ω*_pl_ and $\omega_{{\text{pl}}_{n}}$ , have relations with the *B*-independent coupling strengths as

(8) $$g^{2}=\frac{\omega_{\text{pl}}^{2}d_{\text{QW}}}{\varepsilon_{\text{bg}}L_{\text{eff}}},$$

(9) $$g_{n}^{2}=\frac{\omega_{{\text{pl}}_{n}}^{2}d_{\text{QW}}}{\varepsilon_{\text{bg}}L_{\text{eff}}}.$$

Here, *ɛ*_bg_ = *ɛ*_r_ is the background relative permittivity, *τ* (*τ*_*n*_) is the lifetime of CR (MP_*n*_), *d*_QW_ is the thickness of the quantum well, and *L*_eff_ is the effective cavity length. Using the above relative permittivity, the transmission spectra can be obtained through the transfer-matrix method.

On the other hand, the polariton dispersion can be calculated from the *B*-dependent coupling strengths. The Heisenberg equations for the operators ${\hat{a}}_{+}$ , ${\hat{a}}_{-}^{†}$ , *b*, and *c*_*n*_ form a closed set describing the CR-active polariton modes, while those of ${\hat{a}}_{-}$ , ${\hat{a}}_{+}^{†}$ , *b* ^†^, and $c_{n}^{†}$ describe the CR-inactive modes. The CR-active polariton annihilation operator is given by the Bogoliubov transformation, ${\hat{p}}_{+}=w{\hat{a}}_{+}+y{\hat{a}}_{-}^{†}+x\hat{b}+\sum_{n}x_{n}{\hat{c}}_{n}$ . In the same manner as performed by Ciuti et al. [3], the polariton frequencies and the coefficients are determined by solving the eigenvalue problem of the following matrix:

(10) $$\left(\begin{array}{ccccc} \omega_{0}+D & -D & i\bar{g} & i{\bar{g}}_{1} & \ldots \\ D & -\omega_{0}-D & i\bar{g} & i{\bar{g}}_{1} & \ldots \\ -i\bar{g} & i\bar{g} & \omega_{\text{c}} & 0 & \ldots \\ -i{\bar{g}}_{1} & i{\bar{g}}_{1} & 0 & \omega_{{\text{MP}}_{1}} & \ldots \\ \vdots & \vdots & \vdots & \vdots & \ddots \end{array}\right).$$

**Figure 3:**
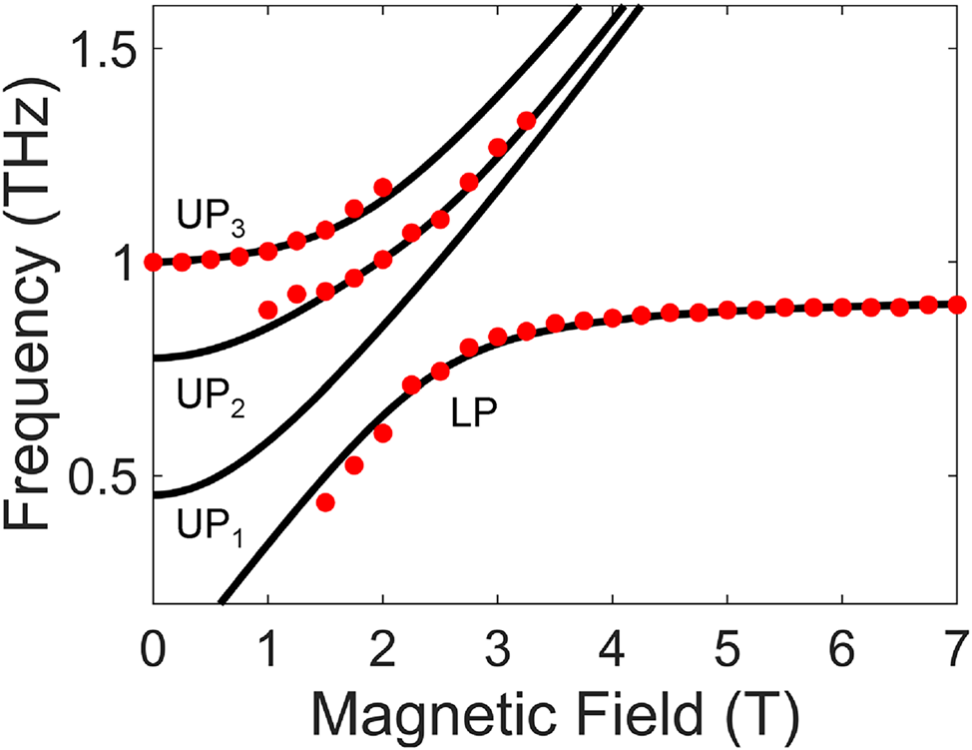
Theoretical analysis of the multimode coupled system. Theoretical polariton frequencies based on the multimode Hopfield model. Red dots are extracted peak frequencies from experiment.

Theoretical transmission spectra for the multimode Landau polariton system, calculated using the transfer-matrix method with the relative permittivity *ɛ*(*ω*) derived in Eq. (7), are shown in Figure 2(b). The resonator structure is simplified to a Fabry–Pérot cavity; details of the calculation setup are provided in the Supplementary Materials. The splitting in the UP branch is observed and captures the key feature of the experimental data. We emphasize that the MP_1_ interacts with the cavity–cyclotron-resonance hybrids at only high frequencies and cannot account for the splitting of the upper polariton at around 1 THz without including the MP_3_ in the model; see Supplementary Materials. The discrepancy in linewidth compared to the experimental spectra is associated with the difference in cavity geometry, with the slots having a reduced *Q*-factor compared to an ideal Fabry–Pérot cavity. From fitting to the experimental peak frequencies, we obtained the normalized coupling strengths of *g*/*ω*_0_ = 0.18 and *g*_1_/*ω*_0_ = *g*_3_/*ω*_0_ = 0.084, confirming the USC of the CR and the SC of MP modes with the cavity mode. Figure 3 shows the calculated polariton frequencies (black solid lines) overlaid with the peak frequencies extracted from the experiments (red dots). Theoretical predictions match the experimental results well. We note that the assumption *g*_1_ = *g*_3_ is generally not valid but is enough to explain the experimental behavior.

**Figure 4:**
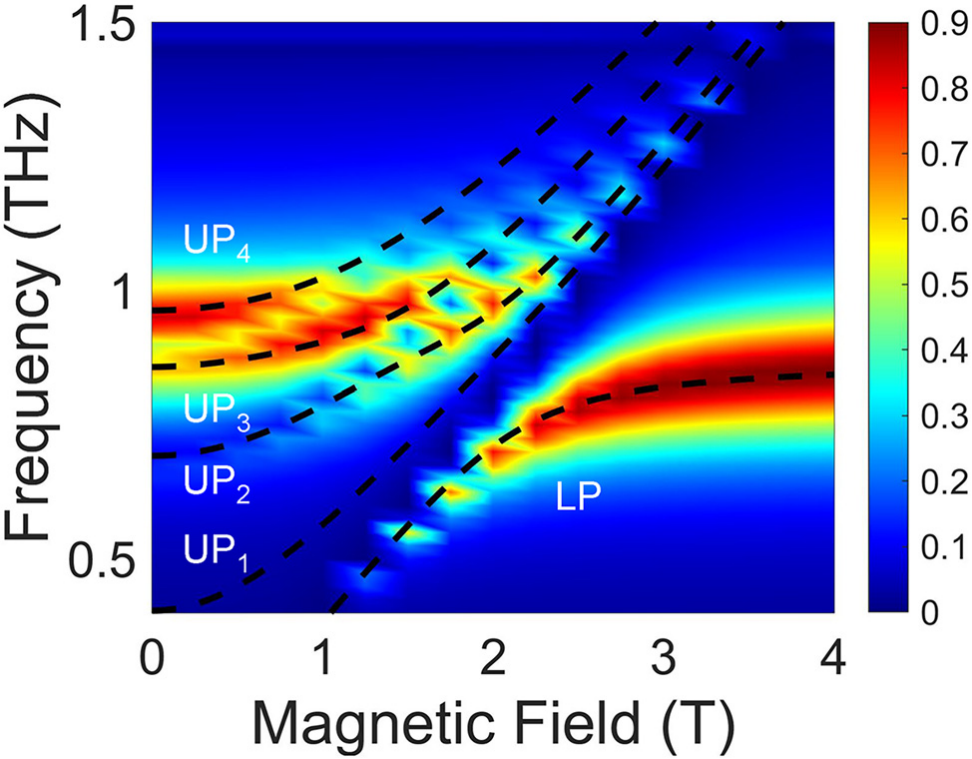
Finite-element simulation of the multimode coupled system. Simulated color map of transmittance as a function of frequency and magnetic field using COMSOL. The black dashed lines are theoretical fits to the polariton frequencies based on the multimode Hopfield model. An additional upper polariton mode, denoted UP_4_, appears due to the influence of the MP_5_ mode.

We further validated our experimental observation and theoretical model using three-dimensional finite-element simulations in COMSOL. Figure 4 shows a color map of transmittance simulated with m* = 0.070*m*_0_ and *ɛ*_r_ = 9.77. Compared with the experimental transmittance in Figure 2(a), the UP branch is split into three parts, separated not only by MP_3_ but also by MP_5_, resulting in UP_4_. Such influence of the MP_5_ could not be observed experimentally due to the weak magnitude. Incorporating the MP_5_ contribution to the multimode Hopfield model, theoretical fits to the polariton frequencies are calculated, shown in black dashed lines in Figure 4; see Supplementary Materials for more detail. The obtained coupling strengths are *g*/*ω*_0_ = 0.13 and *g*_1_/*ω*_0_ = *g*_3_/*ω*_0_ = *g*_5_/*ω*_0_ = 0.062. The slight deviation from the experimentally extracted values likely originates from uncertainties in the sample parameters and the strong effect of the MP_5_ mode. Overall, the simulation is consistent with both the experimental and theoretical analysis of the multimode coupling.

## 3 Discussion

In conclusion, we experimentally explored the coupling between two distinct matter modes mediated by a cavity mode in an ultrastrongly coupled Landau polariton system. Owing to the small-mode-volume slots, cavity photons could interact with both the CR at *k* = 0 and MP modes at finite *k*. Such coexistence of CR and MP excitations has been discussed in previous studies using 2DEG stripes and periodic metasurfaces [20], [30], [33]. However, the multimode interaction demonstrated here could not be accessed due to the excitation geometry and frequency mismatch. In this work, the MP modes affected the Landau polariton through multimode hybridization and led to a splitting of the UP branch. From fitting, we obtained a normalized coupling strength of *g*/*ω*_0_ = 0.18 for CR, and *g*_1_/*ω*_0_ = *g*_3_/*ω*_0_ = 0.084 for MP modes with an in-plane wave vector of *k* = *π*/*d* and *k* = 3*π*/*d*, showing the USC of CR and SC of MP modes to the cavity mode. The experimental results were well explained by the extended multimode Hopfield model and three-dimensional finite-element simulations. Our work establishes a foundation for controlling cavity-mediated nonlocal correlations between matter excitations in the USC regime, unlocking new possibilities for tailoring light–matter interactions.

## Supplementary Material

Supplementary Material Details
