# Supplementary material for: Cavity-mediated coupling between local and nonlocal modes in Landau polaritons
Source: Nanophotonics. 2025 Nov 18;14(25):4647–54. doi: 10.1515/nanoph-2025-0442 (PMC12714039; doi:10.1515/nanoph-2025-0442)
Supplement: Supplementary file 1 — Supplementary Material Details [file j_nanoph-2025-0442_suppl_001.pdf]

Supplementary Materials

S1 Details of the sample fabrication and experimental setup

To fabricate the slots, we utilized a standard photolithography technique to pattern photoresists, followed by Au deposition (50 nm thick) by an electron-beam evaporator on a GaAs substrate containing the QW. After the lift-off process with acetone, we obtained an array of slot cavities (6 mm × 6 mm in lateral dimension), as shown in Fig. 1(a).

Figure S1 shows the THz time-domain magnetospectroscopy setup. The emitted THz waves were guided to be sequentially focused on the samples and the detector by four 90°-off axis parabolic mirrors. The sample was mounted on a 5-mm-diameter aperture. The THz beam diameter at the focal point was about 6 mm, covering the aperture.

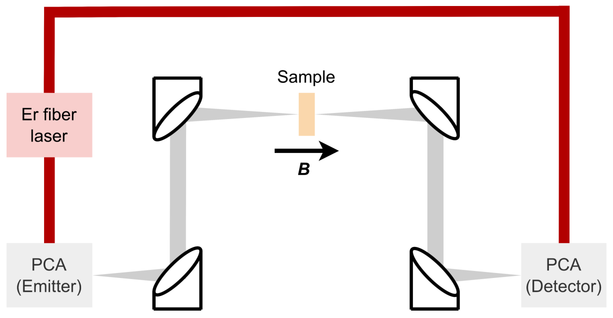

Fig. S1: Experimental setup for THz time-domain magnetospectroscopy measurements.

S2 Details of the theoretical calculation setup

Figure S2 shows the simplified structure of the multimode Landau polariton system adopted in the theoretical calculations. Instead of slot cavities, we simply consider a Fabry–Pérot cavity embedding a 2DEG under a magnetic field. The material parameters are taken from the experiment, with the magnetoplasmon lifetime at 1.2 ps. The GaAs layers have a permittivity of 3.6 and a thickness of $d_{\mathrm{GaAs}} = 22.35\,\mu\mathrm{m}$. The permittivity of the gold layers is calculated using the Drude model. The bare cavity frequency is adjusted to match the experimental peak position at 7 T. The calculated effective cavity length is 84.2 µm. The transmission spectra and the polariton dispersion are obtained with the same parameters.

For fitting to the simulated spectra, d_{GaAs} is set to 23.85 µm, while the other parameters follow those used in the COMSOL simulation. The bare cavity frequency is set to 0.870 THz. The calculated effective cavity length is 58.0 µm.

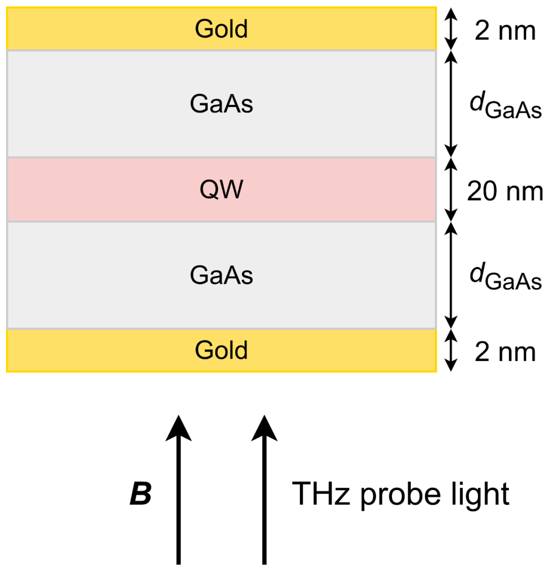

Fig. S2: Schematic of the multimode Landau polariton system used for the theoretical calculations.

S3 Effect of the fundamental MP mode

In our simulation, MP_1 and MP_3 frequencies are predetermined by the material parameters. We considered the higher-order mode (MP_3), because the experimental observation could not be explained by only the interactions between the cavity mode, CR, and MP_1. Fig. S3 shows the theoretical transmission spectra for the Landau polariton system, including only the fundamental MP mode (MP_1). The spectra were calculated using the transfer-matrix method with the relative permittivity $\varepsilon(\omega)$ derived in Eq. (7). We can see that the MP_1 interacts with the other modes at only high magnetic fields at around 3.5 T and cannot account for the splitting of the upper polariton at around 1 THz. Even with lower coupling strengths between the cavity mode and the CR, the anticrossing at around 1.25 T is not possible, because the zero-detuning mag-

netic field of the cavity mode and MP_1 is at 2.18 T, as demonstrated in Fig. 1(c). Therefore, the experimental observation cannot be explained by the coupling between the fundamental mode and the other two modes (CR and the cavity mode), while the model incorporating the higher-order mode reproduces the observed splitting well (Fig. 2(b)).

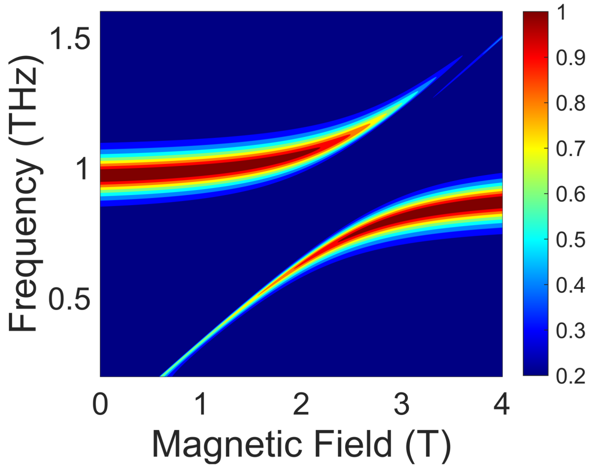

Fig. S3: Theoretical color map of transmittance based on the multimode Hopfield model, including only the $n = 1$ mode.
